# Extracellular MMP-9-Based Assessment of Ocular Surface Inflammation in Patients with Primary Open-Angle Glaucoma

**DOI:** 10.1155/2019/1240537

**Published:** 2019-04-03

**Authors:** Anna Zaleska-Żmijewska, Ewa Strzemecka, Zbigniew M. Wawrzyniak, Jacek P. Szaflik

**Affiliations:** ^1^Department of Ophthalmology, II Faculty of Medicine, Medical University of Warsaw, Żwirki i Wigury 61, 02-091 Warsaw, Poland; ^2^Ophthalmic University Hospital (SPKSO), Warsaw, Poland; ^3^Faculty of Electronics and Information Technologies, Warsaw University of Technology, plac Politechniki 1, 00-661 Warsaw, Poland

## Abstract

**Purpose:**

Objective assessment of dry eye disease (DED) severity and ocular inflammation using the InflammaDry® test for extracellular matrix metalloproteinase-9 (MMP-9) and the impact of antiglaucoma eye drops in people with primary open-angle glaucoma (POAG).

**Methods:**

Overall, 90 adults (180 eyes) were included: 60 had been diagnosed with POAG and were treated with prostaglandin analogue monotherapy and 30 were suspected of having POAG but did not receive any treatment (control group). Of those treated with prostaglandin eye drops, 30 received a preservative-free formulation (tafluprost) and 30 were treated with a formulation containing the preservative benzalkonium chloride (BAK) (latanoprost). Measurement of extracellular MMP-9 levels (InflammaDry test) provided a marker for ocular surface inflammation. Further assessments of disease severity and inflammation comprised Goldmann applanation tonometry for intraocular pressure (IOP), Schirmer's test with anesthesia, ocular surface staining with unpreserved fluorescein (Oxford scale index), tear breakup time (TBUT), McMonnies questionnaire, and the Ocular Surface Disease index (OSDI).

**Results:**

Clinically significant MMP-9 levels (>40 ng/mL) were detected in tear film from 46.7% of subjects treated with BAK-containing medication. In contrast, only 16.7% of subjects treated with preservative-free medication or untreated individuals demonstrated similar MMP-9 levels. This difference was statistically significant (*p* < 0.05). MMP-9 results correlated with other indicators of inflammation and disease severity. BAK-containing medication was associated with rapid TBUT (<5 seconds) in 50% of cases, while only 10% of untreated subjects and individuals using preservative-free medication demonstrated comparable TBUT results.

**Conclusion:**

Measurement of ocular surface MMP-9 level provides a useful marker for inflammation and DED in POAG. Use of a preservative-free topical prostaglandin formulation results in lower levels of ocular inflammation, compared with BAK-containing medication.

## 1. Introduction

Glaucoma is defined as a group of neuropathies that result in progressive optic nerve atrophy, with characteristic changes in the optic nerve head and progressive visual field defects. These changes are usually accompanied by elevated intraocular pressure (IOP), which is the most widely known risk factor of glaucoma. Primary open-angle glaucoma (POAG) may be asymptomatic well into midstage development. Following diagnosis and initiation of topical eye drop treatment, it becomes a chronic and symptomatic disease. In more than 60% of cases, patients treated for glaucoma are also diagnosed with ocular surface disorders (OSDs). Tolerance to different topical therapies varies, and the development of dry eye disease (DED) symptoms during chronic therapy may have considerable impact on life quality and compliance.

Prostaglandin analogues are currently the most frequently used IOP lowering medications [1]. Most eye drops contain a preservative in addition to the active substance. Benzalkonium chloride (BAK) is the most frequently used preservative with the longest history of application. BAK works like a detergent, damaging bacterial cell membranes. Unfortunately, BAK also damages cells within healthy tissues when used chronically. The toxic action of preservatives on the ocular surface has been widely demonstrated *in vitro* and *in vivo*. These adverse effects depend on the accumulation of the BAK dose and can be observed when the preservative is used for a long time, even in low concentrations, as is the case with conservatively treated glaucoma patients. Adverse effects caused by eye drop preservatives are sometimes difficult to identify; they produce unspecific and remote changes. Application of many BAK doses cause changes on the ocular surface and increase inflammatory marker concentrations. Histopathological studies of the conjunctiva have demonstrated inflammation, squamous metaplasia, and subconjunctival fibrosis in the conjunctiva and Tenon's capsule associated with the use of topical preservatives. Some patients may develop irreversible ocular surface changes [1, 2].

BAK decreases the stability of the precorneal tear film. The detergent effect on the lipid layer results in increased evaporation and reduced tear film stability, due to decreased density of goblet cells within the conjunctival epithelium. Symptoms most frequently reported include burning, watery eyes, redness, and blurred vision. Frequency of adverse effects increases with the number of drops administered. Some patients with subjective symptoms of OSDs do not show any obvious characteristic features of inflammation other than subclinical inflammation. It is therefore important in clinical practice to recognize patients who, due to ocular surface inflammation, require either anti-inflammatory treatment or topical immunosuppression with cyclosporin A. This is particularly relevant for patients with planned intraocular surgery (cataract or glaucoma procedures), refractive surgery, and in young patients likely to use glaucoma eye drops for extended periods due to longer life expectancy.

A precise diagnosis of DED may be clinically challenging. Although a number of tests can be used to assess the condition of tear film, both Schirmer's test and tear breakup time (TBUT) often do not correlate with clinical symptoms reported by the patient. Since increased osmolarity is the most characteristic property of tear film in DED, evaluation of tear osmolarity seems to be the most credible diagnostic test. Chotikavanich et al. demonstrated that raised matrix metalloproteinase-9 (MMP-9) levels correlate with severity of dry eye symptoms and TBUT disorders and that it may be the most sensitive indicator in dry eye diagnostics [3].

MMPs are proteolytic enzymes produced by ocular surface and glandular epithelium cells as well as inflammatory cells that proliferate into those tissues [3]. MMPs remodel extracellular matrix and play an important role in the process of wound healing [3]. MMP-9 belongs to the gelatinase family of enzymes, which damage elastin and type IV, V, and VII collagen. MMP-9 is a nonspecific inflammatory marker [4]. It plays a crucial role in splitting of basal membrane components and tight protein junctions, which maintain the corneal epithelial barrier function [3]. The normal MMP-9 level in human tears is low (3–40 ng/ml) [5]. Elevated MMP-9 levels have been found in the tears of patients with DED [3, 6, 7]. Increased ocular surface MMP-9 concentrations after refractive surgery (LASIK and PRK) are responsible for prolonged healing, dry eye symptoms, and haze. They also play an important role in recurrent corneal erosions. Vernal keratoconjunctivitis (VKC) is an ophthalmic disease closely related to elevated MMP-9 levels. Eosinophils and mast cells, which are typically responsible for inflammatory response in allergies, are also key factors in the production of MMP-9. Inflammation at the ocular surface results in corneal changes that are considered frequent and dangerous complications of VKC. Elevated MMP levels have been observed in other corneal diseases such as advanced keratoconus, fungal inflammation, and pterygium. MMP-9 concentration is also elevated in OSDs such as blepharitis with or without concomitant rosacea and in the course of Sjögren's syndrome. However, MMP-9 is not used in diagnostics for these diseases.

Therefore, it seems advisable to develop a simple, inexpensive, and efficient test to assay MMP-9 levels in bodily fluids. InflammaDry® (RPS Diagnostics Inc, Sarasota, USA) is a rapid test that detects elevated MMP-9 concentrations in tear fluid samples taken from the lower eyelid's palpebral conjunctiva. It uses direct sampling microfiltration technology. Results are obtained in 10 minutes with high sensitivity (85%) and specificity (94%) [8]. A positive result indicates MMP-9 levels >40 ng/ml [5]. Other methods used for evaluating ocular surface inflammations are conjunctiva impression cytology and direct tear sampling for immunohistochemical testing. However, they require specialist instruments and a laboratory facility to read the results.

The current study aimed to evaluate the conjunctival expression of MMP-9 in the tear film of people with POAG. Study outcomes were compared for those treated with BAK-containing prostaglandin analogue eye drops and preservative-free prostaglandin analogue eye drops and for a control group comprising individuals receiving no treatment for their POAG. Conventional objective and subjective assessments of DED and ocular surface inflammation were also conducted, and outcomes examined for correlations with the MMP-9 test.

## 2. Materials and Methods

A prospective, unblinded, and single-center study was conducted between March 2016 and May 2016 at the Ophthalmic University Hospital in Warsaw. In total, 90 adults (>18 years) were included in the study and underwent a series of tests to examine objective measures of ocular inflammation and symptomatic disease control. Of these, 60 had been diagnosed with POAG and were assigned to the active treatment groups. The remaining 30 were suspected of having POAG and assigned to the untreated control group. Assessments were conducted during one study visit, and all subjects underwent the same combinations of tests, regardless of the treatment group. Assessments were repeated twice, and outcome values were averaged.

The study protocol was approved by the Bioethical Commission of the Medical University of Warsaw. The study was conducted in accordance with the Declaration of Helsinki. Each subject was provided with written and oral information regarding the study design and objectives, and written informed consent was obtained from all participants.

### 2.1. Study Population

Participants were divided into subgroups according to treatment history. All subjects receiving prostaglandin analogue treatment (preservative-free or BAK-containing formulations) had received treatment for at least one year. Overall, 30 subjects were treated with BAK-containing eye drops (latanoprost), 30 used preservative-free topical medications (tafluprost), and 30 participants received no POAG treatment (control).

### 2.2. Primary Objective: Ocular MMP-9 Levels

Extracellular MMP-9 levels were measured using the commercially available InflammaDry strip test (Rapid Pathogen Screening, Inc, Sarasota, FL, USA). Positive results using this test indicated that tear fluid MMP-9 levels were >40 ng/mL.

### 2.3. Exploratory Objectives: Disease Severity and Ocular Inflammation

Best-corrected visual acuity (BCVA) was assessed, and Goldmann applanation tonometry was used to measure IOP. Cup-to-disc (C/D) ratio fundoscopy was performed using a Volk 78 or Volk 90 lens.

Schirmer's testing with topical anesthesia (5% proparacaine HCl, Alcaine®, Alcon Laboratories Inc., TX, USA) was first conducted. Then, an average TBUT was assessed following application of an unpreserved fluorescein strip soaked with unpreserved physiological salt (0.9%). Finally, ocular surface staining with unpreserved fluorescein (using the Oxford scale index) was done.

Levels of irritation were assessed using the McMonnies scale (Grade 0 to Grade 2) and questionnaire, where Grade 0 indicated no ocular irritation and Grade 2 was strong ocular irritation. The Ocular Surface Disease index (OSDI) questionnaire, comprising 12 questions across 3 subcategories, provided a measure of disease impact on quality of life. Average scores were translated to a 100-point scale, in which 0 represented the least possible impact on quality of life and 100 indicated the greatest impact.

### 2.4. Statistical Analysis

The comparison of mean variables for the study groups and the control group was analyzed in pairs with the *t*-Student test (for normal distribution variables) or the Mann–Whitney *U* test (for other variables). Mean values for all groups were compared with the analysis of variance, or ANOVA (for normal distribution and homogeneous variance), or the Kruskal–Wallis test (for variables with other distributions or nonhomogeneous variance). Variance homogeneity was evaluated with Levene's test and distribution normality with the Shapiro–Wilk and Kolmogorov–Smirnov tests. For material ANOVA values, differences in the mean values were assessed with post hoc Tukey's HSD, Scheffe test, and Bonferroni tests for multiple comparisons.

The adopted significance value of the differences and nonparametric tests was *α* < 0.05, with the test significance value adopted for others.

## 3. Results

### 3.1. Population Demographics

The majority of subjects included in the study were female (80%) (Table 1). Overall, mean age across all study groups was 65.3 years, and the average age of subjects in the active treatment groups (preservative-free and BAK-containing eye drops) was higher than those in the untreated control arm; 67.6 years for the preservative-free treatment group, 67.1 for those treated with BAK-containing eye drops, and 61.2 years for the untreated control group (Table 1). When compared with the control group, the mean age of those receiving active treatment (*n*=60) was significantly greater (67.4 years) (ANOVA *p*=0.019, post hoc tests *p* < 0.04). There were no statistically significant differences concerning mean BCVA across study groups (Table 1).

### 3.2. Ocular MMP-9 Levels

Ocular MMP-9 testing revealed that 46.7% of subjects treated with BAK-containing medication demonstrated clinically significant levels of MMP-9 (>40 ng/mL) (Table 2). In contrast, just 16.7% of subjects in each of the other groups (untreated and preservative-free) showed positive results when tested for elevated levels of MMP-9 (Table 2). This difference was statistically significant (*p*=0.0125).

### 3.3. Disease Severity and Inflammatory Assessments

Statistically significant differences were observed regarding mean IOP values between the preservative-free treatment group and the untreated group (Tukey's HSD test, *p*=0.009) (Table 3). Statistically significant differences were also demonstrated concerning the mean IOP value across both active treatment groups (preservative-free and BAK-containing; *n*=60), compared with the untreated control group, 12.5 mmHg versus 13.5 mmHg, respectively.

Mean C/D ratios also revealed significant differences between active treatment groups versus the untreated control group (*n*=60), Tukey's HSD and Scheffe tests *p*=0.001 (Table 3).

TBUT results correlated with MMP-9 measures for each of the treatment groups. In total, 50.0% of subjects using BAK-containing medication and 10.0% of those receiving preservative-free or no treatment reported TBUT of <5 seconds. This difference was statistically significant (*p* < 0.05).  Table 4 shows results for other measures of DED. Schirmer's test showed a significant difference between those treated with BAK-containing medication (8.27 mm/5 min) compared with untreated subjects (11.63 mm/5 min) and individuals receiving preservative-free medication (12.07 mm/5 min) (*p* < 0.05) (Table 4). Fluorescein staining of the conjunctiva and cornea (graded using the Oxford index) was greater in the BAK-containing treatment group, compared with the untreated and preservative-free groups (Table 4).

In total, 70% of subjects in the control group and 43.3% in the preservative-free group reported Grade 0 scores using the McMonnies scale. In contrast, just 26.7% of subjects in the BAK-containing treatment group scored ocular irritation at Grade 0. Grade 2 scores were reported by 26.7% of the BAK-containing treatment group, 10% of the preservative-free group, and 6.7% of the untreated control group.

OSDI questionnaire results concerning the impact of DED on the quality of life showed those using preservative-free treatments rated their condition as having the lowest impact, compared with untreated subjects and individuals treated with BAK-containing medication (Table 5). However, these differences were not significant (Levene's test, *p*=0.218; ANOVA, *p*=0.374).

## 4. Discussion

MMP-9 testing provided an accurate indication of the presence and level of ocular inflammation associated with different approaches to POAG treatment. MMP-9 test results correlated with conventional objective measures of DED such as TBUT (Goodman–Kruskal's Gamma correlation coefficient equals −0.622, *p* < 0.05), Schirmer's test (Goodman–Kruskal's Gamma correlation coefficient equals 0.468, *p* < 0.05), and corneal or conjunctival fluorescein staining indicating that MMP-9 assessments may offer a simple diagnostic tool that supports appropriate treatment selection. Our results also demonstrated that BAK-containing eye drops were associated with raised levels of MMP-9, and therefore surface inflammation and/or damage, compared with preservative-free prostaglandin analogue formulations.

Tear film dysfunction in glaucoma patients is primarily a result of the chronic use of preserved eye drops. The adverse effect of BAK, particularly on the ocular surface, has been well documented [9]. The arrival of preservative-free formulations raised hope for reducing the adverse effect on the ocular surface in patients who require a long-term therapy. Ocular surface changes connected with chronic use of eye drops may be a cause of the patient's lower quality of life, dissatisfaction with the treatment and, in consequence, decreased efficacy of the treatment.

All patients included in this study were previously treated with monotherapy with a formulation of the same therapeutic group (i.e., prostaglandin analogue), eliminating the potential contribution of the active substance to ocular surface inflammation.

The problem of OSDs has been raised in a number of global publications. Disorders of any of the tear film layers result in ailments that are widely defined as the DED. Multiple tests are available that assess functions of the respective tear film layers. These include the TBUT test, Schirmer's test, staining of the ocular surface with fluorescein, lisamine or Rose Bengal, assessment of the conjunctival irritation, and questionnaire assessing subjective problems as reported by the patient (OSDI). However, none of these tests can determine whether the ocular surface is affected by inflammation or simply a lacrimation disorder. DED is frequently associated with subclinical inflammation. Detecting this inflammation and introducing anti-inflammatory or immunosuppressive treatment may significantly improve the ocular surface condition and alleviate symptoms. Results of tear film tests in our study confirmed that glaucoma patients treated with preserved eye drops developed OSDs more often. According to various publications, as many as 65% of chronically treated glaucoma patients have disorders of the ocular surface [10].

At present, a standardized protocol is not in place to detect OSDs in glaucoma patients. Subjective symptoms are assessed with the use of the OSDI questionnaire developed for dry eye patients. However, results achieved in glaucoma patients may be biased by the patient's subjective feelings related to glaucomatous visual field damage [11].

The current study showed that abnormal TBUT results (<5 seconds) were achieved in 50.0% of the subjects using BAK-containing eye drops and in just 10% of those using preservative-free prostaglandin and in 10.0% of untreated individuals. Similar results were achieved by Walimbe et al. who demonstrated that switching from a BAK-containing formulation to preservative-free topical medication led to TBUT improvements [9]. Other studies have also confirmed that Schirmer's test results improved, following a switch from a preserved medication to a BAK-free formulation [12]. All participants in the current study were diagnosed with disorders of the aqueous layer of the tear film, which were the most intense in the group BAK-containing treatment group.

The McMonnies scale was used to assess the degree of ocular irritation. The higher the grade, the stronger the irritation. A higher proportion of subjects indicated that they had no ocular irritation in the preservative-free group (43.3%) compared with the BAK-containing treatment group (26.7%). Grade 2 scores, associated with significant conjunctival irritation, were reported by a higher proportion of subjects receiving BAK-containing treatment (26.7%), compared with those using preservative-free eye drops (10.0%). These results are consistent with previous studies showing that the degree of conjunctival hyperemia is reduced following a switch to a BAK-free formulation [12].

Conjunctival and corneal staining with fluorescein was significantly greater in subjects treated with BAK-containing medication compared with those using preservative-free treatment. Again, these results are aligned with previous studies that found a higher Oxford index score in patients using more BAK-containing eye drops [12]. Superficial punctate epitheliopathy may be present in as many as 50% of patients treated with BAK-containing polytherapy [13].

TBUT scores and Oxford scale results in the current study are consistent with those reported in similar studies, which also found that Schirmer's test and OSDI scores did not differ between the active treatment groups and untreated control groups [14]. We observed that all subjects showed OSDs, which were assessed through OSDI scores as mild (preservative-free and untreated groups) and moderate (BAK-containing treatment group) DED. In contrast, Walimbe et al. demonstrated that a switch from a BAK-containing formulation to a preservative-free medication resulted in a significant decrease in OSDI scores from 18.06 to 7.06 [9]. One should remember, however, that this is a solely subjective assessment. Other studies have demonstrated comparable Schirmer's test and OSDI results with the current study [15–18].

The majority of established quantitative assays for ocular inflammation require invasive techniques (conjunctival or corneal biopsy) or complex laboratory tests, whereas the MMP-9 test is minimally invasive and simple to conduct in clinical practice. Although other authors have described using the MMP-9 test in patients after refractive surgery procedures with DED and pseudoexfoliation syndrome, our study allowed the effects of chronic glaucoma treatment on proinflammatory biomarkers to be examined [5, 19, 20]. Almost half of subjects (46.7%) using BAK-containing eye drops had MMP-9 tear levels of >40 ng/mL, while only 16.7% of the patients in the other two groups tested positive for MMP-9. Previously, impression cytology analysis of HLA DR expression and IL-6, IL-8, and IL-10 interleukin levels in flow cytometry showed that HLA DR antigen expression was significantly higher in those treated with preserved formulations compared with patients treated with preservative-free 0.5% timolol or placebo/no treatment [21]. In addition, interleukin levels were elevated in all glaucoma patient groups compared with the group of healthy subjects [21]. Impression cytology has also shown that higher concentrations of inflammatory markers are present in patients using glaucoma drops compared with control groups [13].

Small studies have assessed the effect of POAG and pseudoexfoliation glaucoma on concentrations of MMPs and their inhibitors (TIMPs) in the conjunctiva. Elevated expression of MMPs and TIMPs was highlighted in both types of glaucoma compared with the healthy subjects [22]. These results are aligned with the current study.

An impression cytology examination of conjunctival epithelium cells of rabbits treated with a prostaglandin formulation for at least 1 year showed elevated levels of IL-1*β* and IL-6 interleukins and MMP-1, MMP-3, and MMP-9 matrix metalloproteinases [23]. At the same time, the rabbits showed decreased levels of TIMP-1 and TIMP-2 matrix metalloproteinase inhibitors in tears [24]. Immunohistochemical staining demonstrated also an elevated expression of MMP-1 and MMP-9 along with a decreased expression of TIMP-1 in the rabbits' corneal stroma, confirming the hypothesis regarding the presence of inflammation in eyes chronically treated with preserved drops [23]. These results are also consistent with the results published in 2010 by Honda et al., demonstrating elevated MMP-1 and MMP-9 concentrations in patients treated with BAK-preserved latanoprost drops for 8 weeks compared with untreated subjects (91.2 ng/mL and 19.7 ng/mL in the control group; *p* < 0.001) [24].

MMP-9 testing appears to provide a robust marker for ocular inflammation and symptoms of DED associated with POAG. Further studies may be required in larger patient groups to establish the usefulness of this approach in routine clinical practice.

## 5. Conclusion

Assessment of tear film MMP-9 levels provides a simple and effective diagnostic approach that supports identification of ocular inflammation and DED in people with POAG. Ocular surface MMP-9 levels are higher in people treated with BAK-containing eye drops compared with those receiving preservative-free treatment, suggesting the levels of ocular inflammation is increased with preserved topical therapies. Preservative-free medication has a favorable effect on the ocular surface while ensuring IOP control.

## Figures and Tables

**Table 1:** Summary of demographic characteristics and BCVA by study group.

	Preservative-free treatment (tafluprost) (*n*=30)	BAK-containing treatment (latanoprost) (*n*=30)	Untreated (control) (*n*=30)	Total (*n*=90)
*Age*				
Mean ± SD	67.6 ± 7.8	67.1 ± 8.7	61.2 ± 11.9	65.3 ± 10.00
Range	50–90	50–78	31–81	31–90

% Females	73.3	86.7	80	80

*BCVA, right eye*				
Mean ± SD	0.85 ± 0.19	0.81 ± 0.25	0.90 ± 0.20	0.85 ± 0.21

*BCVA, left eye*				
Mean ± SD	0.84 ± 0.23	0.80 ± 0.19	0.90 ± 0.15	0.85 ± 0.20

SD, standard deviation.

**Table 2:** Ocular MMP-9 status by study group.

	Preservative-free treatment (tafluprost) (*n*=30)	BAK-containing treatment (latanoprost) (*n*=30)	Untreated (control) (*n*=30)
MMP-9 (>40 ng/mL)			
Negative	25 eyes (83.3%)	16 eyes (53.3%)	25 eyes (83.3%)
Positive^*∗*^	5 eyes (16.7%^*∗*^^,‡^)	14 eyes (46.7%)	5 eyes (16.7%^*∗*^^,‡^)

Level of significance: ^*∗*^*p* < 0.05; ^‡^significance of difference relative to the BAK-containing treatment (latanoprost) group.

**Table 3:** IOP and C/D ratios in each study group.

	Preservative-free treatment (tafluprost) (*n*=30)	BAK-containing treatment (latanoprost) (*n*=30)	Untreated (control) (*n*=30)	Total (*n*=90)
IOP (mmHg) mean ± SD				
C/D ratio	12.00^*∗∗*^^‡^ ± 1.53	12.40 ± 1.50	13.60^*∗∗*^^‡^ ± 2.88	12.67 ± 2.16
Mean ± SD	0.74^*∗∗∗*^^‡^ ± 0.10	0.69^*∗∗∗*^^‡^ ± 0.10	0.50^*∗∗∗*^^‡^ ± 0.16	0.64 ± 0.16

SD, standard deviation. ^*∗∗*^*p* < 0.01; ^*∗∗∗*^*p* < 0.001; ^‡^significant difference relative to control group.

**Table 4:** Evaluation of dry eye syndrome using Schirmer test, conjunctiva, and cornea fluorescein staining.

	Preservative-free treatment (tafluprost) (*n*=30)	BAK-containing treatment (latanoprost) (*n*=30)	Untreated (control) (*n*=30)	Total (*n*=90)
*Conjunctiva staining score*				
0	25 (83.3%)	18 (60.0%)	23 (76.7%)	—
1	5 (16.7%)	9 (30.0%)	7 (23.3%)	—
2	0 (0%)	3 (10%)	0 (0%)	—

*Corneal staining score*				
0	29 (96.7%)	23 (80.0%)	28 (93.3%)	—
1	1 (3.3%)	5 (16.7%)	2 (6.7%)	—
2	0 (0%)	2 (3.3%)	0 (0%)	—

Schirmer's test (mm/5 minutes)	12.07^*∗*^^†^ ± 5.80	8.27 ± 4.67	11.63^*∗*^^†^ ± 7.64	10.66 ± 6.32

Level of significance: ^*∗*^*p* < 0.05; ^†^significance of difference relative to the BAK-containing treatment (latanoprost) group.

**Table 5:** Mean OSDI values by study group.

	Preservative-free treatment (tafluprost) (*n*=30)	BAK-containing treatment (latanoprost) (*n*=30)	Untreated (control) (*n*=30)	Total (*n*=90)
OSDI score ± SD	17.15 ± 13.21	23.00 ± 18.80	21.62 ± 17.81	20.57 ± 16.79

SD, standard deviation.

## Data Availability

The data used to support the findings of this study are available from the corresponding author upon request.
